# Population genomic analysis of the rice blast fungus reveals specific events associated with expansion of three main clades

**DOI:** 10.1038/s41396-018-0100-6

**Published:** 2018-03-22

**Authors:** Zhenhui Zhong, Meilian Chen, Lianyu Lin, Yijuan Han, Jiandong Bao, Wei Tang, Lili Lin, Yahong Lin, Rewish Somai, Lin Lu, Wenjing Zhang, Jian Chen, Yonghe Hong, Xiaofeng Chen, Baohua Wang, Wei-Chiang Shen, Guodong Lu, Justice Norvienyeku, Daniel J. Ebbole, Zonghua Wang

**Affiliations:** 1 State Key Laboratory of Ecological Pest Control for Fujian and Taiwan Crops, College of Plant Protection, Fujian Agriculture and Forestry University, Fuzhou, 350002 China; 2 Fujian-Taiwan Joint Center for Ecological Control of Crop Pests, Fujian Agriculture and Forestry University, Fuzhou, 350002 China; 3 Department of Plant Pathology and Microbiology, National Taiwan University, Taipei, Republic of China; 4 Department of Plant Pathology and Microbiology, Texas A&M University, College Station, TX USA; 5 Institute of Ocean Science, Minjiang University, Fuzhou, 350108 China

## Abstract

We examined the genomes of 100 isolates of *Magnaporthe oryzae* (*Pyricularia oryzae*), the causal agent of rice blast disease. We grouped current field populations of *M*. *oryzae* into three major globally distributed groups. A genetically diverse group, clade 1, which may represent a group of closely related lineages, contains isolates of both mating types. Two well-separated clades, clades 2 and 3, appear to have arisen as clonal lineages distinct from the genetically diverse clade. Examination of genes involved in mating pathways identified clade-specific diversification of several genes with orthologs involved in mating behavior in other fungi. All isolates within each clonal lineage are of the same mating type. Clade 2 is distinguished by a unique deletion allele of a gene encoding a small cysteine-rich protein that we determined to be a virulence factor. Clade 3 isolates have a small deletion within the *MFA2* pheromone precursor gene, and this allele is shared with an unusual group of isolates we placed within clade 1 that contain *AVR1-CO39* alleles. These markers could be used for rapid screening of isolates and suggest specific events in evolution that shaped these populations. Our findings are consistent with the view that *M. oryzae* populations in Asia generate diversity through recombination and may have served as the source of the clades 2 and 3 isolates that comprise a large fraction of the global population.

## Introduction

*Magnaporthe oryzae* (*Pyricularia oryzae*), the causal agent of rice blast disease, results in significant yield losses worldwide and is an important threat to food security [1, 2]. Although deployment of resistance genes is an important strategy for disease control, the genetic diversity of the pathogen has limited the effectiveness of this approach. Understanding the structure of the current population and how diversity is generated is important for managing the disease.

Like other heterothallic ascomycetes, *M. oryzae* has two mating types determined by a single master locus carrying either Mat1-1 or Mat1-2 sequences [3, 4]. Although sexual reproduction in the field has not been observed directly, population studies indicate that sexual recombination occurs in geographical regions associated with the origin of rice domestication [5, 6]. However, there are also examples where isolates of both mating types are present but recombination appears to be absent or very rare [7–9]. In addition, *Magnaporthe* may experience parasexual recombination in the field [10–14]. Transposable elements also contribute to generating diversity through transposition to cause gene deletion or otherwise inactive genes [15, 16]. Relationships between populations adapted to different hosts have been defined using multiple gene loci and recently with population genomic analysis [16–20]. These studies are critical for understanding current population structures and the potential for invasion of new hosts, such as wheat [21].

Population studies conducted on *M. oryzae* rice isolates found that local *M. oryzae* populations are often dominated by isolates of one mating type [7, 22–26], suggesting that rice blast populations are predominately clonal lineages that are selected based on the host genotype, such that collections made from any one location may show restricted genetic diversity.

In studies using microsatellite markers [6, 27], three main genetic clusters were found in *M. oryzae* population. One genetically diverse population undergoes sexual recombination and could be separated into two subgroups, each containing both mating types. The two other genetic clusters were mainly clonal and these groups were distributed globally, but not uniformly so. A recent study also identified recombining and clonal populations in six lineages, and estimated the time of divergence of the lineages from each other approximately 1000 years before present (YBP) [28].

Understanding the complexity of these populations will require additional genome and population analyses to define the relationships within and between clades with more resolution and to help identify, from among the evolutionary events that have accompanied the expansion of particular lineages, which genes were targeted by natural selection. Here we describe high-quality sequencing, de novo assembly, and annotation for globally distributed *M. oryzae* isolates to assess mechanisms that may help explain the observed population structure. We investigated the population structure of isolates from China as well as several different regions around the world and assigned isolates into three major clades. Clade 1 contained isolates of both mating types, clade 2 contained only Mat1-2 isolates, and clade 3 contained only Mat1-1 isolates. We were unable to detect gene flow between the two single-mating-type clades. Clade 2 contained a deletion of a gene encoding an intracellular effector that was able to suppress BAX-mediated cell death in *Nicotiana benthamiana*. The occurrence of genetically distinct clonal lineages indicates that hybrids between clades have reduced fitness and/or that mating between clades is restricted. To search for variation in the genes of the mating pathway that may contribute to poor fertility, we surveyed allelic variation in genes known to be involved in fungal mating pathways. This analysis revealed a fixed non-synonymous substitution allele of *MST20* in clade 3. Clade 3 isolates also possess a 30 bp deletion within the *MFA2* pheromone precursor gene. These unique alleles can serve as markers to assign isolates to clades. We estimate the clades diverged with each other approximately 1000 YBP. Additional genomic diversity within the clades revealed that rapid genetic divergence is occurring between the two clades undergoing asexual reproduction, which appear to have undergone expansion ~250–400 YBP.

## Materials and Methods

### Isolate collection, cultivation, DNA preparation, genome sequencing, and annotation

Isolates in this study are described in supplemental Table S1 [18, 19, 29–34]. To maximize genetic diversity, the isolates were selected based on distinct geographic location and year of collection. Genomic DNA was isolated as previously described [19]. Sequencing libraries were prepared using the Illumina Paired-End DNA sample Prep Kit and sequenced using an Illumina Hiseq2500. De novo sequence assembly was conducted with CLC Genomic Workbench 7.0. Gene predictions were conducted through a combination of evidence-based prediction by Exonerate (version 2.2.0) [35], and ab initio gene prediction with Fgenesh from SoftBerry (http://linux1.softberry.com/berry.phtml). Evidence-based and ab initio gene prediction results were combined by EVidenceModeler [36].

### Single-nucleotide polymorphism discovery, identification of mating-related genes, and transposon element annotation

Sequenced reads were aligned to the reference genome (70-15 v8) with Bowtie2 [37, 38]. SAMtools (version 0.1.19) and Genome Analysis Toolkit (GATK, version 3.3-0), for single-nucleotide polymorphism (SNP) calling with allele/depth <0.9, and filtered for multiple alleles with other parameters set to the default [39–41]. The genome-to-genome SNP calling was performed with MUMmer, version 3.23 (-maxmatch -c 100 –p) [42]. Amino-acid sequences of yeast mating-related genes were obtained from the *Saccharomyces* Genome Database (http://www.yeastgenome.org/). Yeast homologs were identified by reciprocal BLASTP (*E* < 10^−5^) with the *M. oryzae* genome [3, 43].

### Phylogenomic tree construction, population structure, and divergence time estimation

The phylogenomic tree was built using FastTree (Version 2.1.9) based on the SNP dataset with the approximately-maximum-likelihood model [44]. STRUCTURE analysis was conducted using Structure 2.3.4 with whole-genome SNPs, and *K* value selected by the Evanno method in STRUCTURE HARVESTER [45, 46]. Principal component analysis (PCA) based on SNPs was conducted by using Tassel 5.0 and plotted with R [47]. BEAST (v1.8.3) was used to estimate divergence time with the timescale calibrated using the collection year of samples (1978-2015) and two *Setaria viridis* isolates as outgroup [48]. We used the HKY + Gamma substitution model and selected the strict clock model based on prior test of both strict and relaxed clock results analyzed with Tracer (v1.6), and Markov chain Monte Carlo analyses of 10 million iterations were run in duplicate to check for convergence. The timescaled tree was displayed with FigTree (v1.4.3, http://tree.bio.ed.ac.uk/software/figtree/). Additional public sequence data (GenBank PRJNA245782) were assessed for concordance with our analysis of the main group of genomes.

### Population genetics and genomics

DNAsp was used for nucleotide diversity calculations [49]. KaKs_Caculator 2.0 was used to calculate Ka/Ks values with the YN model [50, 51]. Consensus genome sequences obtained with the SNP dataset were used for recombination rate (*ρ*) calculation with CPLDhat as well as nucleotide diversity, Tajima’s *D*, and Theta calculation with VariScan [52, 53]. Vcftools (v0.1.12b) was used to calculate *F*_st_ values with a 50 kb window [54]. To calculate *F*_st_ value for genes, SNPs located in gene regions were used for calculations in Vcftools. Gene presence and absence variation (PAV) was detected by searching the 70-15 gene sequence against each isolate with BLASTN (*E* < 10^−10^).

### Live-cell imaging analysis and *Agrobacterium*-mediated transient expression assay for suppression of plant cell death response in *N. benthamiana*

A 2 kb region of upstream sequence along with the coding region for *MGG_17227* (Guy11) was fused with mCherry:nuclear localization signal (NLS) or green fluorescent protein (GFP) for cloning into the pKNTG vector. *M. oryzae* Guy11-transformed strains were used for rice leaf sheath inoculation and transformants with strong localization to the biotrophic interface complex (BIC) were documented with a Nikon NiE confocal microscope [55].

Agroinfiltration was performed as described with modifications [56, 57]. The coding region of MGG_17227 with or without the signal peptide predicted by SignalP4.1 was cloned into pCXSN with primers listed in supplemental Table S4 [58, 59]. MGG_17227, AvrPiz-t, and vector control strains were infiltrated into 5-week-old *N. benthamiana* leaves. After 24 h, plants were again infiltrated with *Agrobacterium* strains carrying the BAX expression clone. The infiltrated plants were kept at 22–25 °C with a 16 h light/8 h dark period for 3–4 days until cell death symptoms induced by BAX were detected. Leaves in which both positive and negative controls responded appropriately were then assessed for suppression of BAX-induced cell death.

## Results

### Phylogenomic characterization of rice-infecting *M. oryzae* populations

**Fig. 1:**
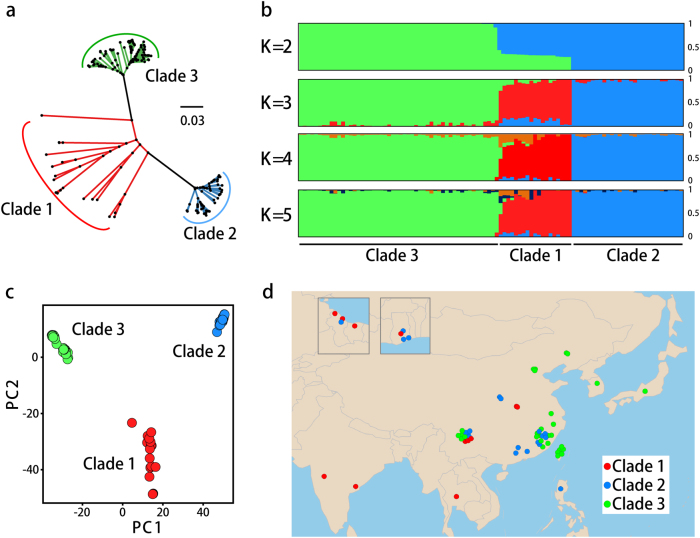
Phylogenetic relationship of *M. oryzae* isolates.** a** Phylogenomic tree of *M. oryzae* isolates based on whole-genome SNP data. **b** STRUCTURE analysis of *M. oryzae* isolates. Each color in the plots represents the cluster membership coefficients. The presence of several colors in the same strain suggests admixture. **c** Principal component analysis (PCA) of *M. oryzae* isolates. **d** Origin of samples in this study. Red, blue, and green dots represent isolates of clade 1, clade 2 and clade 3. Some dots represent more than one isolate with same collection site. Suriname, French Guiana (left), and Ghana (right) are presented separately in two windows, for more information see Table S1

To characterize rice-infecting populations, we sequenced 88 rice isolates from different geographical locations with 32- to 104-fold coverage (average ~60-fold). We also acquired genome sequences of 12 previously published isolates, and thereby increased our sample size to 100 (supplemental Table S1). We used a genome-to-genome SNP calling approach because the sequencing reads for 10 out of 12 previously published genomes sequences were not available. A total of 751.6K SNPs combined from the 100 genomes covering 58.4K sites of the reference genome were identified, which represents about ~0.15% of the genome. The phylogenomic tree showed that the samples could be partitioned into three clades with two clades (clade 2 and clade 3) showing well-defined lineages that diverged from a common ancestral population, whereas the third clade (clade 1) contained more diversified samples from multiple lineages (Fig. 1a). To further validate the categorization of these isolates, we conducted PCA and STRUCTURE analysis. Consistent with phylogenomic result, both PCA and STRUCTURE analysis also categorized the isolates into three clades (Fig. 1b, c). *K* = 2 to *K* = 5 summary plots for samples indicate the most consistent number of genetic populations was *K* = 3 as determined using the Evanno method (Fig. 1b). Population studies by others showed that genetic lineages observed in populations of *M. oryzae* field isolates are globally distributed and our collection, includes isolates from Asia, Africa, and South America, is consistent with this global pattern (Fig. 1d).

To further test the population structure, we performed reads-to-genome SNP calling for the 90 isolates with available reads by mapping sequencing reads to reference genome and then used the corresponding results to construct a phylogenomic tree followed by PCA and STRUCTURE analysis. Reads-to-genome analyses yielded 954K SNPs covering 72.6K sites of the reference genome, and the results obtained with these SNPs are consistent with results obtained with genome alignment SNPs (Supplemental Fig. S1abc).

### The divergence of *M. oryzae* field populations shows correlation with mating type

Reproduction isolation constitute major factors that contribute to population divergence, we therefore examined the reproduction machinery in our *M. oryzae* population to search for an imbalance that may provide evidence of clonality [60]. We found that the phylogenomic partitioning of isolates into three clades strongly correlated with the mating type pattern of the isolates (Table 1) and that all clade 3 isolates are Mat1-1 mating types, while all isolates in clade 2 are Mat1-2. Clade 1 consisted of both Mat1-1 and Mat1-2 isolates, including isolates from Yunnan, Hubei, Suriname, Guiana, Thailand, India and Ghana (Supplementary Table S1).

**Table1:** Mating type of *M. oryzae* in three clades

Clade	Mat1-1	Mat1-2	*n*
Clade 1	8	11	19
Clade 2	0	29	29
Clade 3	52	0	52

*n*: number of isolates in each clade

### Origin and divergence of *M. oryzae* field populations

**Fig. 2:**
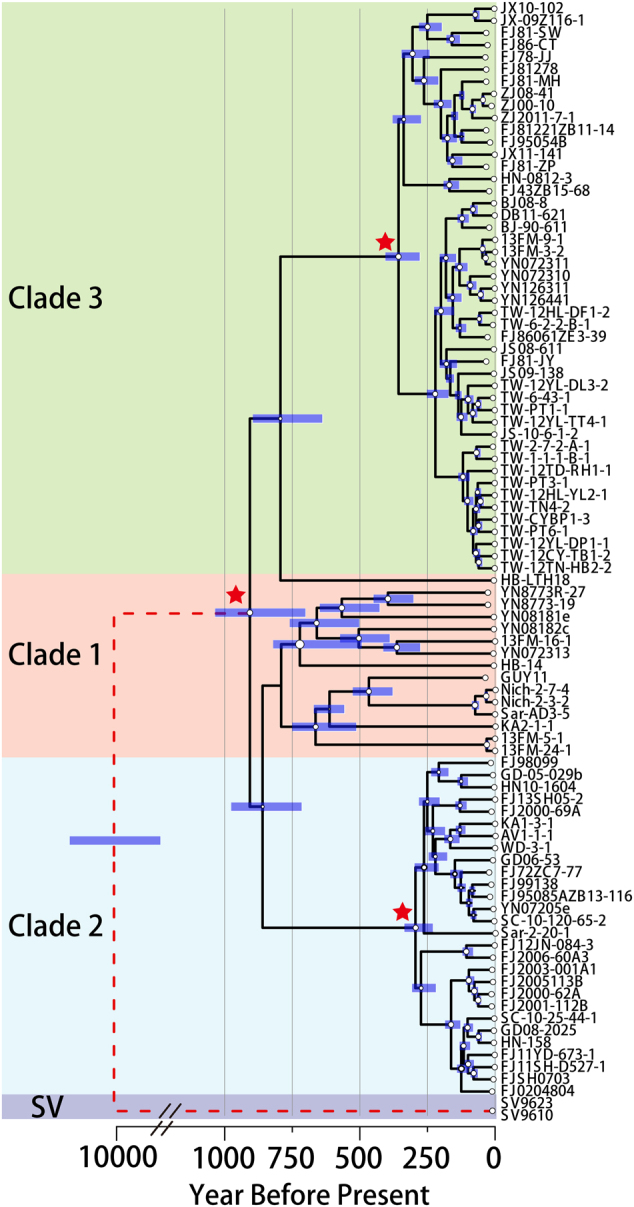
Estimation of divergence time of *M. oryzae* populations. Chronogram from Bayesian phylogenetic analysis on whole-genome SNP dataset. Whole-genome SNP sequences were calibrated by collection dates. Blue bars indicate 95% highest posterior density intervals of node-age estimates. SV represent isolates collected from *S. viridis*. The red dashed line shows the break in the time axis of SV to rice population branch. Red stars marked divergence (star 2) or expansion (stars 1 and 3) time of clades

To trace back the evolutionary history of the *M. oryzae* population, we estimated divergence time of the three clades using *S. viridis* isolates (SV, wild relative of rice isolates) as the outgroup (Fig. 2). This analysis showed that *M. oryzae* rice isolates and SV isolates diverged from each other 8000–12,000 YBP, coincident with rice domestication [20, 61]. Our analysis revealed that *M. oryzae* populations diverged at about ~1000 YBP. Unlike the genetically diverse clade 1, clades 2 and 3 appear to have expanded as clonal lineages at a similar time, just ~250–400 YBP.

**Fig. 3:**
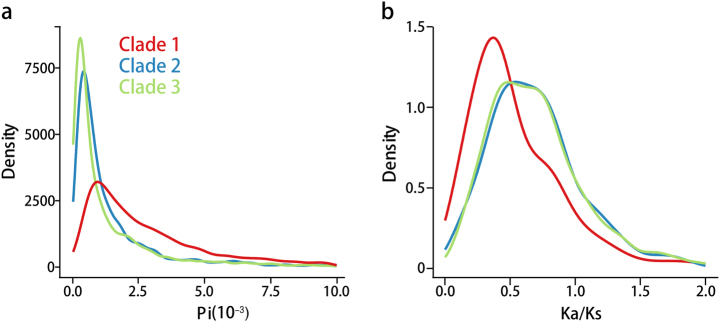
Genomic divergence of *M. oryzae* field populations.** a** Distribution of Pi values against the corresponding density of genes for three clades. **b** Distribution of Ka/Ks values against the corresponding density of genes for three clades. The red, blue, and green colored lines represent the number of genes of clade 1, clade 2, and clade 3, respectively

To further characterize the difference between these three populations, we examined gene PAV between isolates within the three clades. We identified a total of 13,573 PAV events in all isolates and found clade 2 and clade 3 exhibit similar levels of gene deletion. Our analysis showed that the telomere regions are hot spots for gene deletion events (Supplemental Fig. S2). Further comparison of nucleotide diversity (*π*) and Ka/Ks value for genes in each clade yielded differential patterns with clade 1 displaying higher levels of *π* value with lower levels of Ka/Ks (Fig. 3a, b). Clade 2 and clade 3 isolates showed similar patterns of *π* and Ka/Ks values, which were distinct from clade 1.

**Fig. 4:**
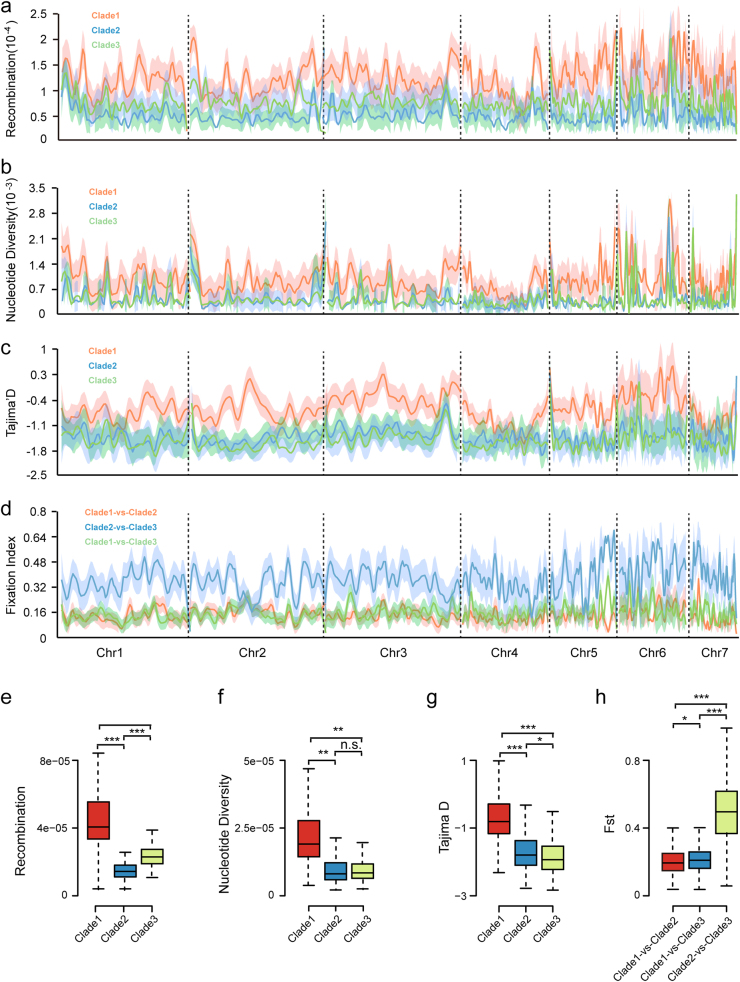
Recombination rate (*ρ*), nucleotide diversity (*π*), and Tajima’s *D* for the three clades of *M. oryzae*.** a**–**d** Whole-genome distribution of recombination rate (*ρ*), nucleotide diversity (*π*), Tajima’s *D*, and *F*_st_ on chromosome I ~ VII of *M. oryzae* with 50 kb windows. **e**–**h** Boxplot of recombination rate, Pi, Tajima’s *D*, and *F*_st_ values of three clades. The Tukey whiskers indicate 1.5 times the interquartile range from the 25th and 75th percentiles. n.s. represents no significance, * represents *p*-value smaller than 0.05, ** represents *p*-value smaller than 1e-5, *** represents *p*-value smaller than 1e-10

Meanwhile, we observed the three clades of isolates showed similar patterns of whole-genome distribution of SNPs (Supplemental Fig. S3). However, intra-clade estimates of whole-genome nucleotide diversity (*π*), Tajima’s *D*, recombination rate (*ρ* = 2*N*_e_*r*), and inter-clade fixation index (*F*_st_) exhibited significant variations between the three clades (Fig. 4 and Supplemental Table S3). Measures of nucleotide diversity and recombination rate for clade 1 (*π* = 3.47 × 10^−4^, *ρ* = 4.36 × 10^−5^) were significantly higher than those of clade 2 (*π* = 1.88 × 10^−4^, *ρ* = 1.54 × 10^−5^) and clade 3 (*π* = 1.87 × 10^−4^, *ρ* = 2.65 × 10^−5^). Low recombination rate is accompanied with low nucleotide diversity as well as significant negative value of Tajima’s *D* (−0.96, −1.81, and −1.93 for clade 1, clade 2, and clade 3, respectively). The pair-wise *F*_st_ value in clade 2 vs. clade 3 (*F*_st_ = 0.40) is also elevated compared with that in clade 1 vs. clade 2 (*F*_st_ = 0.14) and clade 1 vs. clade 3 (*F*_st_ = 0.15), indicating gene flow is absent between clade 2 and clade 3 as expected for non-recombining lineages.

### Identification of divergence-associated genes

**Fig. 5:**
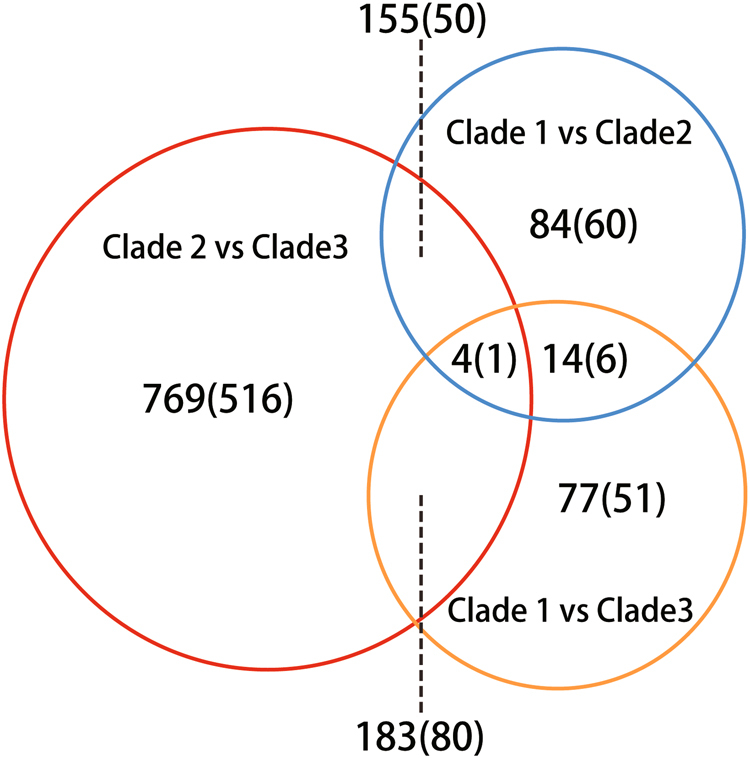
Venn diagram of genes with *F*_st_ > 0.8 in the three clades. Venn diagram depicting the pair-wise comparison of genes with *F*_st_ > 0.8 between the three clades. Numbers in brackets represent the number of genes with non-synonymous substitutions

We searched for genes with *F*_st_ > 0.8 between different clades to identify genes with differential distribution of alleles that might contribute to adaptation or differences in fertility. Such genes may represent the occurrence of divergent selection in gene regions between clades or be the result of fixation of relatively rare alleles at the time of divergence of the lineages. We found 769 genes in the analysis of clade 2 vs. clade 3, among which 516 genes are with non-synonymous substitutions (Fig. 5). Among these 516 genes, we further identified 11 genes that have *F*_st_ > 0.8 in clade 2 vs. clade 3 (Table 2) but <0.3 in clade 1 vs. clade 2 or clade 1 vs. clade 3. We reasoned that such genes might represent alleles that were not selected against in clade 1, but could be adaptive in a clade-specific context or the result of the bottleneck associated with a clonal lineage. The biological annotation of these 11 genes showed 2 of the genes were involved in the pheromone response pathway, another 5 genes involved in metabolic or cellular pathways, and 4 genes with unknown function.

**Table 2:** Annotation of genes with low (<0.3) *F*_st_ in clade 1 vs. clade 2 and clade 1 vs. clade 3, and high (>0.8) in clade 2 vs. clade 3

ID	Yeast ortholog	*F*_st_			Description	GO
		Clade 1 vs. clade 2	Clade 1 vs. clade 3	Clade 2 vs. clade 3		
MGG_00731	FAR11	0.27	0.22	1.00	Required for hyphal anastomosis; protein involved in recovery from cell cycle arrest; acts in response to pheromone	GO:1903436, regulation of mitotic cytokinetic process
MGG_01846	APL6	0.29	0.20	1.00	Transport of alkaline phosphatase to the vacuole via the alternate pathway	GO:0045184, establishment of protein localization
MGG_02874	KOG1	0.04	0.09	1.00	WD repeat-containing protein mip1; subunit of TORC1	GO:0019222, regulation of metabolic process
MGG_03254	MDR1	0.24	0.09	1.00	TBC1 domain family member 10A; cytoplasmic GTPase-activating protein; regulation of Golgi secretory function	GO:0043169, cation binding
MGG_04563	NA	0.27	0.18	1.00	NA	GO:0046872, metal ion binding
MGG_05466	NA	0.29	0.20	1.00	NA	NA
MGG_08984	OPT2	0.29	0.20	1.00	Oligopeptide transporter	GO:0051234, establishment of localization
MGG_10558	NA	0.08	0.15	0.87	NA	NA
MGG_10887	NA	0.23	0.09	0.97	NA	NA
MGG_13215	NA	0.07	0.30	1.00	NA	NA
MGG_13576	SRM1	0.27	0.24	1.00	RCC1 domain-containing protein; nucleotide exchange factor for Gsp1p; suppressor of the pheromone response pathway	GO:0008168, methyltransferase activity

Isolates from single-mating-type lineages are generally of low female fertility. We thus examined sequence variation of mating pathway genes in addition to the two we identified above. We observed 173 SNPs or InDels in 28 genes, and most of the mating pathway-related components were conserved or contained variation that was present across all three clades. For example, we observed a synonymous substitution in *MGG_05199* (*STE50*) for isolates in all three clades (Supplemental Table S2). However, in *MGG_12821* (*MST20*), we identified 53 SNPs/InDels and one of these is a T485A non-synonymous mutation found exclusively in all isolates of clade 3. Amino acid 485 lies between the BEM1-binding motif and the kinase domain [62]. In addition, the pheromone precursor (*MFA2*) gene, *MGG_07733*, was associated with a 30 bp deletion (hereafter full-length allele: type I; 30 bp deletion allele: type II) at position 79 bp of its open reading frame sequence in all clade 3 isolates. Interestingly, the *MFA2* type II allele also appears in a clade 1 lineage (Supplemental Fig. S4) consisting of four isolate sequences deposited at NCBI (isolates 2303.1, 1106.2, K96-07, and K98-10) (Supplemental Fig. S4b). In contrast to clade 3 Mat1-1 isolates, these four isolates were Mat1-2 and also contained a non-functional J-type allele of *AVR1-CO39* [63, 64].

### Absence of a putative effector gene defines clade 2 isolates

**Fig. 6:**
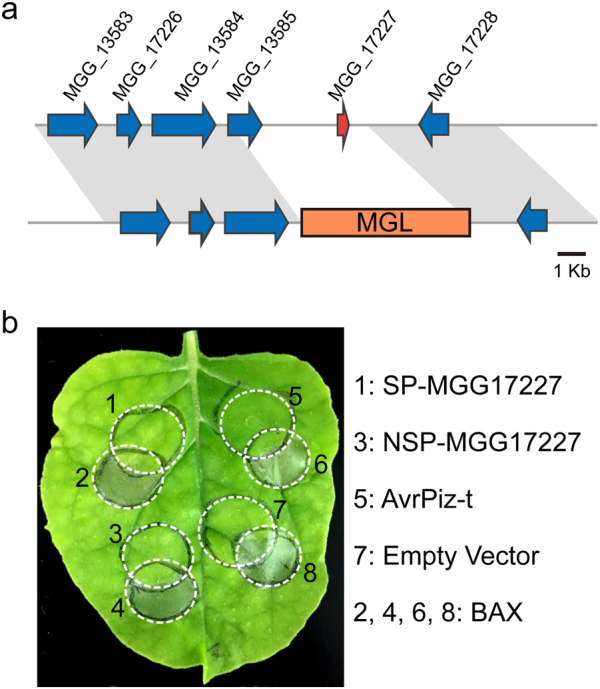
Deletion of a putative effector gene defines clade 2 isolates.** a** Schematic of *MGG_17227* in all of clade 2 isolates indicating *MGG_17227* was replaced by a 5977 bp LINE retrotransposon MGL from 3757 bp upstream to 758 bp downstream of the coding region. **b** MGG_17227 suppresses BAX-induced cell death following *Agrobacterium*-mediated transfection of tobacco leaves. Both MGG_17227 full-length (1) and secretion signal-deleted construct (3) can suppress BAX-induced cell death (2, 4, 6, 8). AvrPiz-t (5) and empty vector (7) have been used as positive and negative controls, respectively

We identified a small, cysteine-rich secreted protein (MGG_17227) that was absent in all clade 2 isolates. The end points of the deletion show sequence identity to the LINE retrotransposon MGL, showing a gene replacement by MGL in all clade 2 isolates replacing sequence from 3757 bp upstream to 758 bp downstream of the coding region. This 4515 bp region includes part of a second hypothetical protein of 303 amino acids (Fig. 6a). The *MGG_17227* gene was shown to be expressed *in planta* but not *in vitro* (Supplemental Fig. S5a). To assess localization *in planta*, a GFP fusion was constructed using the native promoter. The GFP fusion was localized to the BIC during infection of rice (Supplemental Fig. S5b). A second fusion to mCherry containing a NLS was used, and localization to the BIC and to the plant nucleus confirmed that the protein was delivered into the plant cytoplasm (Supplemental Fig. S5c).

To test for a potential role for MGG_17227 to act as an effector, we cloned the coding region with and without the signal peptide for testing in *N. benthamiana* [58]. AvrPiz-t is an effector that suppresses cell death mediated by BAX and was used as a positive control (Fig. 6b) [56]. The MGG_17227 constructs with and without the signal peptide also displayed inhibition of BAX-mediated cell death.

## Discussion

*M. oryzae* rice-infecting isolates from different geographic locations could be grouped into three major clades as shown in our analysis and a former study based on microsatellites markers [6]. The times of divergence could be estimated by taking into account the year of isolate collection and the SNPs between isolates. Based on our own, and a recent study with an independent set of rice isolates [28], these three main groups diverged from each other approximately 700–1000 years ago. Clade 1 genetic diversity is inferred to have expanded from a small population at that time. It was difficult by this analysis to resolve if clades 2 and 3 were derived from clade 1 or were parallel lineages that had diverged earlier from a common ancestral population that later gave rise to clade 1. Based on Supplemental Fig. S4b, the grouping we defined as clade 1 may contain as many as five lineages.

Clades 2 and 3 appear to have diversified recently, within the past ~250–400 years. This is consistent with the recent findings of Gladieux et al. [28] and they attribute these expansions to events in the history of rice cultivation. We found an effector gene, *MGG_17227*, that was absent in all clade 2 isolates. Within clade 1, the closely related isolate pair 13FM-16-1 and YN072313 share a different deletion event that eliminates *MGG_17227* as does a second closely related pair, 13FM-5-1 and 13FM-24-1. This indicates that isolates lacking this effector can persist in clade 1 and clade 3 populations. For effectors that are recognized by plant resistance genes (also known as avirulence genes), gene PAV is common [65]. Conceivably, *MGG_17227* may also be recognized as an avirulence gene and deletion of the gene led to the expansion of the clade, perhaps following the movement of cultivars with a corresponding resistance gene. If so, clade 2 isolates transformed with *MGG_17227* could provide a useful tool in screening for such a resistance gene. The *MGG_17227* allele should allow for simple initial screening to identify clade 2 isolates by amplification with appropriate primers to distinguish the clade 2 replacement allele.

The view that clonal evolution is associated with clade 2 and clade 3 is supported by lack of clear recombination events within the genome sequences. To search for a potential genetic barrier to mating, we examined genes involved in fungal mating pathways. *F*_st_ comparison identified two mating pathway-related genes, orthologous to *Saccharomyces cerevisiae* Far11 and Smr1, containing non-synonymous substitution alleles displaying high *F*_st_ values in the clade 2 vs. clade 3 comparison. We observed a unique non-synonymous substitution allele of another *S. cerevisiae* mating pathway ortholog, *MST20* [62], fixed in clade 3. Clade 3 was also associated with a 30 bp deletion in the *MFA2* locus. Further study will be needed to determine whether these alleles impact mating behavior.

The *MFA2* gene is expressed in a mating-type-specific manner and should not be expressed in the Mat1-1 isolates of clade 3, but would be expressed in Mat1-2 isolates [43]. Interestingly, the *MFA2* type II allele is also present in a Mat1-2 clade 1 lineage of four isolates that also uniquely contain non-functional *AVR1-CO39* J-type alleles [63, 64]. Couch et al. assessed populations of *M. oryzae* from different hosts and found 70 *Panicum* isolates clustered within the rice pathogen clade. The *Panicum* isolates contained *AVR1-CO39* homology by hybridization, as did the most closely related rice isolate [20], which we assume is a member of the group described here. They also proposed that a rice pathogen population invaded *Panicum* as a new host. Notably, the *Panicum* isolates in that study are not pathogenic to rice. Analysis of *Panicum* isolate* AVR1-CO39* alleles would help determine if this was simply a host shift from rice to *Panicum*, in which case one would expect to see the J-type allele in *Panicum* isolates.

The occurrence of the clade 3 *MFA2* type II allele in this group is also interesting. We speculate that either the *MFA2* type II allele existed prior to divergence of the clades, which involved recombination along the path for at least one of the lineages, since the two lineages are of opposite mating type, or that the occurrence of the clade I *MFA2* type II allele is a result of a rare recombination of clades 1 and 3. Detection of the *MFA2* allele type and mating type may serve as a simple means to initially screen for members of clade 3 and this clade 1 lineage.

Our study indicates that the divergence time of *M. oryzae* rice isolates from the closely related population infecting *S. viridis*, corresponded with the time of domestication of rice [20, 28]. As has been noted by others [27, 28], we noticed that most isolates collected from japonica rice-growing regions such as Beijing, Japan, Korea, Liaoning, Taiwan, and Fujian (in the 1980s), are grouped together in clade 3 and most isolates collected from indica rice-growing regions, such as Fujian since the 1990s are grouped in clade 2. The Fujian Province example indicates a shift from clade 3 to clade 2 isolates as the predominant members of the pathogen population was driven by a host genotype shift. Clade 1 contains isolates mostly have been sampled from regions that are associated with the cultivation of both japonica and indica, including Yunnan, India, and Thailand [66]. This observation further supports earlier research showing that japonica and indica rice cultivars exhibit differential susceptibility and resistance toward different isolates within the *M. oryzae* population [67–70].

Consistent with the view that clades 2 and 3 represent clonal lineages, our analyses found little to no evidence of recombination within clades 2 and 3, reduced allelic diversity, and lower genotypic diversity between isolates within the two clades compared to clade 1. The *F*_st_ value between clade 2 and clade 3 is higher than that between either clonal lineage with clade 1. This highlights the high genetic differentiation between the ancestral isolates leading to the clade 2 and 3 clonal lineages.

Although all clades are globally distributed, local populations may be composed predominantly of isolates from a single clade. It has been recognized that lineage exclusion may be a strategy for deployment of resistance genes with enhanced durability [71, 72]. Thus, if lineage exclusion of clade 2 isolates could be employed in indica rice-growing areas (or clade 3 isolates in japonica rice-growing areas), the less-well-adapted clades would be able to cause disease, but severity may be reduced. However, areas where clade 1 isolates are present indicate the potential for enhanced recombination, therefore, determining the clades represented within local populations will provide useful information for breeding programs. The screening of the *MGG_17227*, *MFA2*, and Mat loci could help to quickly distinguish between clades (supplemental Table S5).

In the collection of isolates we analyzed, we found that a significant fraction of pathogen isolates are members of the two clonal clades that have spread globally. Clade 1 is a genetic reservoir that could generate new lineages to adapt to new host genotypes while it maintains a sufficient level of success to avoid being outcompeted by clonal lineages. It will be of interest to determine if a similar genetic structure occurs for *M. oryzae* populations parasitizing other hosts.

## Electronic supplementary material

Supplemental Figures(PDF 1300 kb)

supplemental Table S1(XLSX 23 kb)

supplemental Table S2(XLSX 2042 kb)

supplemental Table S3(XLSX 13 kb)

supplemental Table S4(XLSX 11 kb)

supplemental Table S5(XLSX 23 kb)
