# Supplementary material for: Population genomic analysis of the rice blast fungus reveals specific events associated with expansion of three main clades
Source: ISME J. 2018 Mar 22;12(8):1867–78. doi: 10.1038/s41396-018-0100-6 (PMC6051997; doi:10.1038/s41396-018-0100-6)
Supplement: Supplementary file 1 — Supplemental Figures(PDF 1300 kb) [file 41396_2018_100_MOESM1_ESM.pdf]

Population genomic analysis of the rice blast fungus reveals specific events associated with expansion of three main clades

Running title: Population genomics of *Magnaporthe oryzae*

This work was supported by grants from the Natural Science Foundations of China (U1305211, 31770156, 91231121, 31401692), the National Key Research and Development Program of China (2016YFD0300700), the Fujian Province 100 Talent Program, USDA NIFA Hatch project 1013944, China Scholarship Council, and the Scientific Research Foundation of the Graduate School of Fujian Agriculture and Forestry University.

Zhenhui Zhong[a,b]*, Meilian Chen[a,b]*, Lianyu Lin[a,b], Yijuan Han[a,b], Jiandong Bao[a,b], Wei Tang[a,b], Lili Lin[a,b], Yahong Lin[a,b], Rewish Somai[a,b], Lin Lu[a,b], Wenjing Zhang[a,b], Jian Chen [a,b], Yonghe Hong[a,b], Xiaofeng Chen[a,b], Baohua Wang[a,b], Wei-Chiang Shen[c], Guodong Lu[a,b], Justice Norvienyeku[a,b#], Daniel J. Ebbole[a,d#], Zonghua Wang[a,b,e#]

[a] State Key Laboratory of Ecological Pest Control for Fujian and Taiwan Crops, College of Plant Protection, Fujian Agriculture and Forestry University, Fuzhou 350002, China.

[b] Fujian-Taiwan Joint Center for Ecological Control of Crop Pests, Fujian Agriculture and Forestry University, Fuzhou, 350002, China.

[c] Department of Plant Pathology and Microbiology, National Taiwan University.

[d] Department of Plant Pathology and Microbiology, Texas A&M University, College Station, TX, USA.

[e] Institute of Ocean Science, Minjiang University, Fuzhou,350108, China.

* These authors contributed equally to this work.

# To whom correspondence should be addressed. Email: wangzh@fafu.edu.cn, Phone: +86-13706948783, d-ebbole@tamu.edu, Phone: +1-9798454831, jk_norvienyeku@fafu.edu.cn Phone: +86-13174505261.

Keywords: population genomics, population divergence, mating type, asexual reproduction

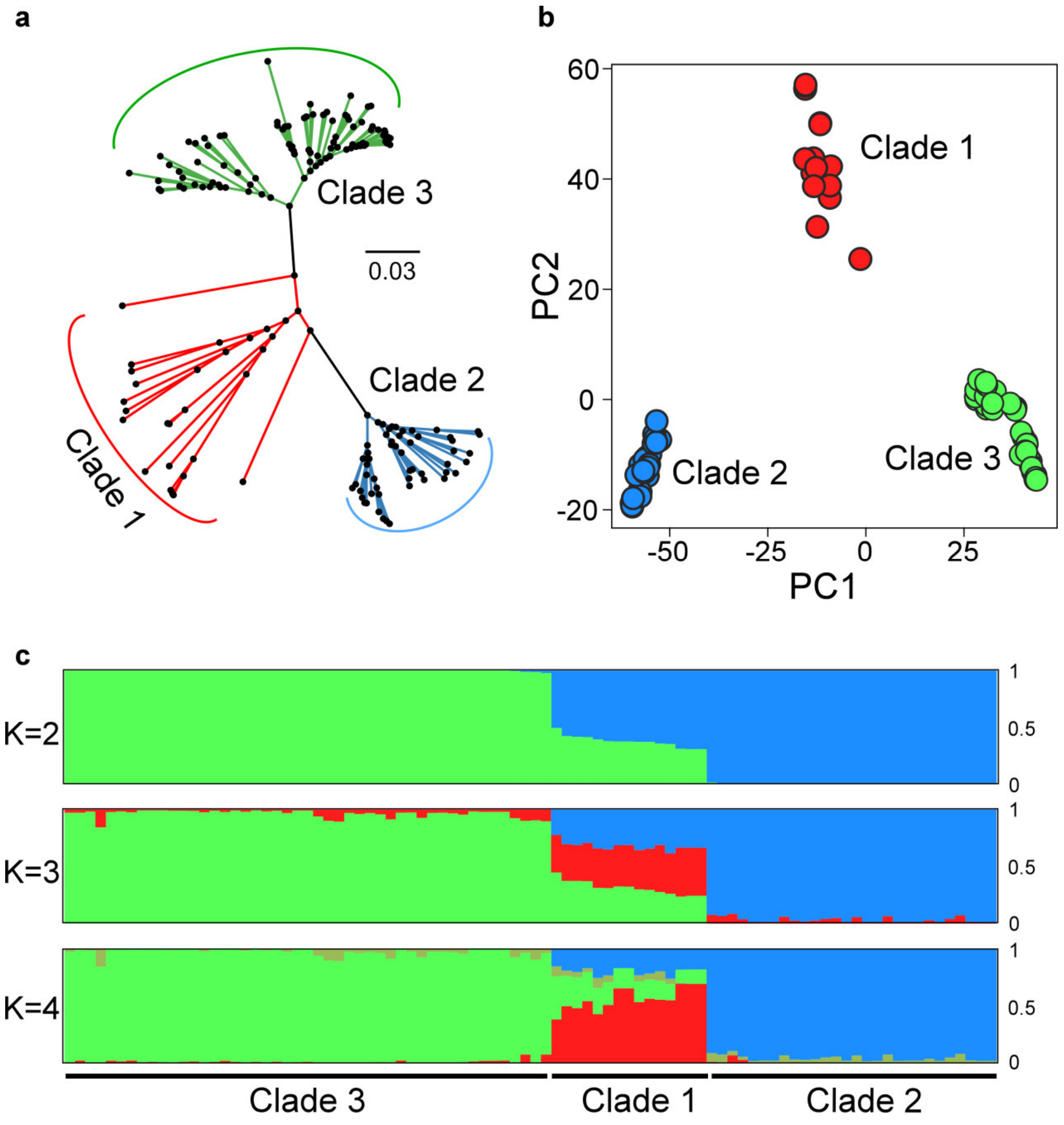

Supplemental Fig. S1 Phylogenetic relationship of 90 isolates of *Magnaporthe oryzae* with SNPs obtained from GATK. a Phylogenomic tree of *M. oryzae* isolates based on whole genome SNPs data. **b** Principal Component Analysis (PCA) of *M. oryzae* isolates. **c** STRUCTURE analysis of *M. oryzae* isolates. Each color in the plots represents the cluster membership coefficients. The presence of several colors in the same strain suggests admixture.

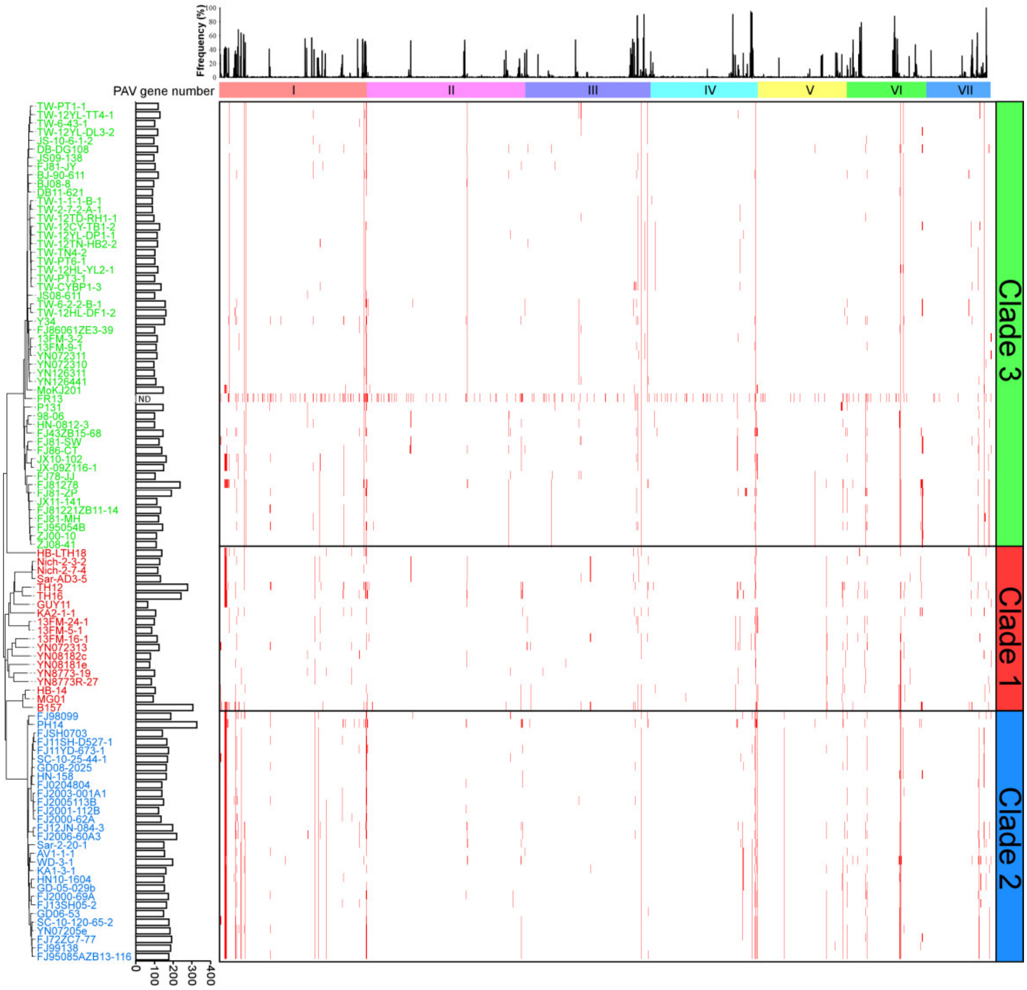

Supplemental Fig. S2 Genome-wide visualization of gene Present and Absent Variation (PAV) profiles in three clades. Phylogenetic tree and total number of genes with PAV were present at left. Distribution of PAVs on chromosomes were marked with red for each isolate. Frequency of each gene in all isolates were calculated and present with line plot at the top. The FR13 isolate was excluded in this study since it has 1 015 gene of PAV, which is an obvious deviation with other isolates, whose numbers ranging from 63 to 326.

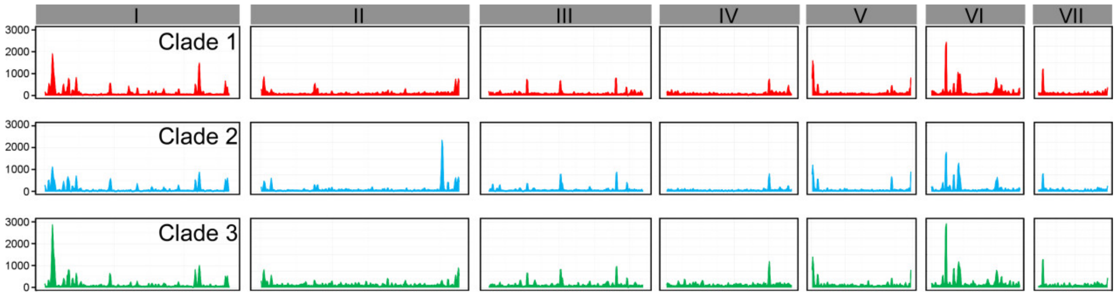

Supplemental Fig. S3 Genome wide SNPs distribution. Visualization of genome wide distribution of SNPs density of clade 1, clade 2 and clade 3 in chromosome I ~ VII with 50 kb windows.

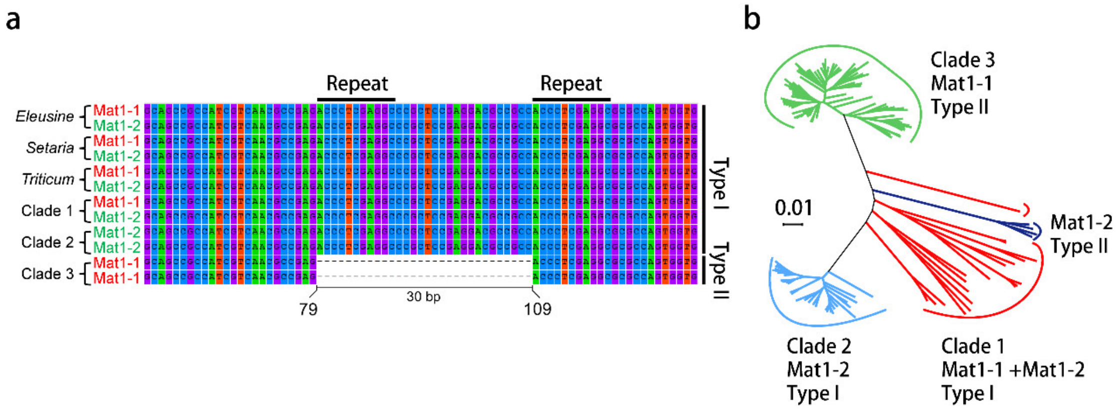

Supplemental Fig. S4 Occurrence and phylogenetic distribution of the MFA2 deletion allele. a Nucleotide alignment of MFA2 reveals an 11 bp repeat sequence associated with the site of a 30 bp deletion. *Eleusine*, *Seteria* and *Triticum* are isolates from *E. indica*, *S. viridis*, *T. aestivum*. Type I and Type II are alleles with or without 30 bp deletion at position 79 in the coding region of MFA2. **b** Phylogenomic tree incorporation analysis of strains 2303.1, 1106.2, K96-07 and K98-10 that make up the clade marked Mat1-2 Type II. This clade contains the MFA2 30 bp deletion.

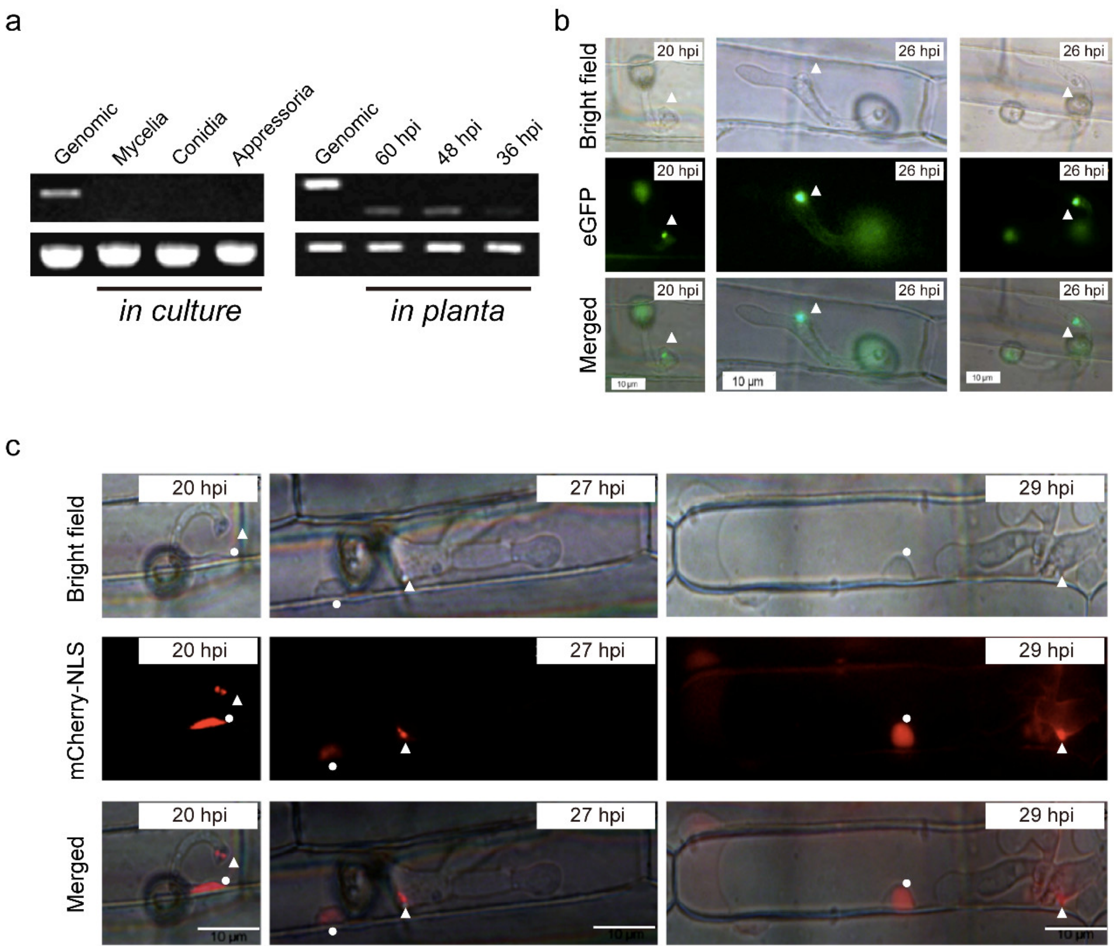

Supplemental Fig. S5 Expression and *in planta* localization of MGG_17227. a Expression pattern of MGG_17227 in mycelia, conidia, and appressoria, 36 hpi, 48 hpi and 60 hpi. **b** *In planta* localization of MGG_17227::GFP at 20 and 26 hpi. **c** *In planta* localization of MGG_17227::mCherry-NLS at 20, 27 and 29 hpi. White triangles present Biotrophic Interface Complex (BIC) and white dots present rice nucleus. Bar = 10 µm.
